# Phytomycobiomes and Ecosystem Services: Mechanisms, Evidence and Routes to Application

**DOI:** 10.3390/jof12010001

**Published:** 2025-12-19

**Authors:** Rizwan Ali Ansari, Kobilov Ergash Egamberdievich, Madjidova Tanzila Raximovna, Yarmatova Dilbar Sa’dinovna, Belyalova Leylya Enverovna, Aminjonov Sharifkul Abbasovich, Abdullayev Davlat Muqumovich, Tukhtaev Mustafa Kurbonovich

**Affiliations:** 1 Department of Ecology and Life Safety, Faculty of Geography and Ecology, Samarkand State University named after Sharof Rashidov, Samarkand 140104, Uzbekistan; 2 Department of Physiology and Biochemistry of Humans and Animals, Samarkand State University named after Sharof Rashidov, Samarkand 140104, Uzbekistan; 3 Department of Dermatology and Venereology, Samarkand State Medical University, 18 Amir Temur Street, Samarkand 140104, Uzbekistan

**Keywords:** fungal diversity, soil health, sustainable forestry, climate resilience, microbial characterization

## Abstract

Phytomycobiomes refer to the fungal consortia that inhabit plant tissues and the rhizosphere. Their documented functions include nutrient mobilization, carbon retention, stress mitigation and pathogen suppression, although measurable effects often depend on plant and soil conditions. In this review, we examine the current evidence for their ecological relevance and assess the molecular approaches most commonly used to characterize them. Arbuscular Mycorrhizal (AM) fungi, endophytes and saprotrophic taxa indicate measurable gains in nutrient acquisition, disease resistance and soil aggregation, although long-term consistency is rarely evaluated. Each function appears to have an explicit mechanistic attribution, with direct links between fungal groups, enzymatic pathways and measurable ecosystem outcomes. Several sequencing-based techniques are available, yet none offer complete accuracy. Internal Transcribed Spacer (ITS) amplicon surveys provide rapid taxonomic coverage but suffer from primer bias; shotgun metagenomics offers functional insight but at significant financial cost; and quantitative polymerase chain reaction (qPCR) assays remain useful for targeted quantification, whereas long-read technologies show promise but still lack widespread adoption. The field faces a number of unresolved constraints, including limited knowledge of host range, inconsistent performance under fluctuating environmental conditions and the absence of a standardized bioinformatic pipeline. Despite these limitations, we regard phytomycobiomes as viable candidates for replacing or reducing synthetic inputs, provided their application is guided by context-specific evidence rather than broad generalization.

## 1. Introduction

Fungal communities (mycobiomes) are essential in promoting ecological balance, plant and soil health. Gillevet et al. [1] first coined the term ‘mycobiome’ to refer to the fungal component of the microbiome of any specific location or host. Phytomycobiomes comprise fungal communities that link plant function to ecosystem processes [2,3]. This component decomposes organic matter, regulates nutrient cycling, and also helps maintain trophic balance in terrestrial and aquatic ecosystems [4]. Functional groups such as white-rot and brown-rot fungi have distinct biochemical processes with an effect on nutrient release and the stabilization of soil organic matter [5,6]. Fungal necromass and polymers form a major fraction of stable soil organic matter, while mycorrhizal associations affect plant–decomposer competition and nutrient flow [7]. Phytomycobiomes inhabit soils, decomposing residues, living tissues and aquatic niches, where saprotrophic fungi dominate and degrade cellulose, lignin and chitin through oxidative and hydrolytic (cellulases, hemicellulases, laccases and peroxidases) enzymes [8,9,10,11]. These fungi regulate nutrient dynamics and plant performance, but their community structure varies with host traits, nutrient status and microbial interactions [12].

**Figure 1:**
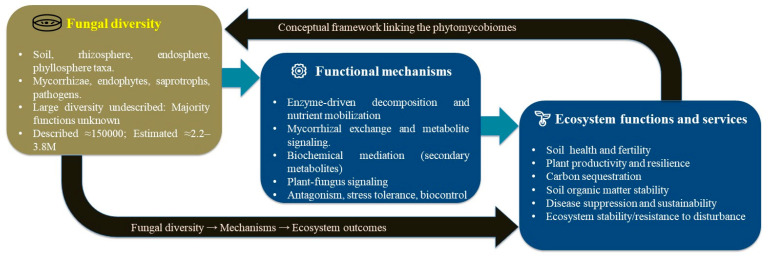
A conceptual framework illustrating the interrelationships between fungal diversity [34,35,38], its principal functional mechanisms, and the resultant ecosystem outcomes.

**Figure 2:**
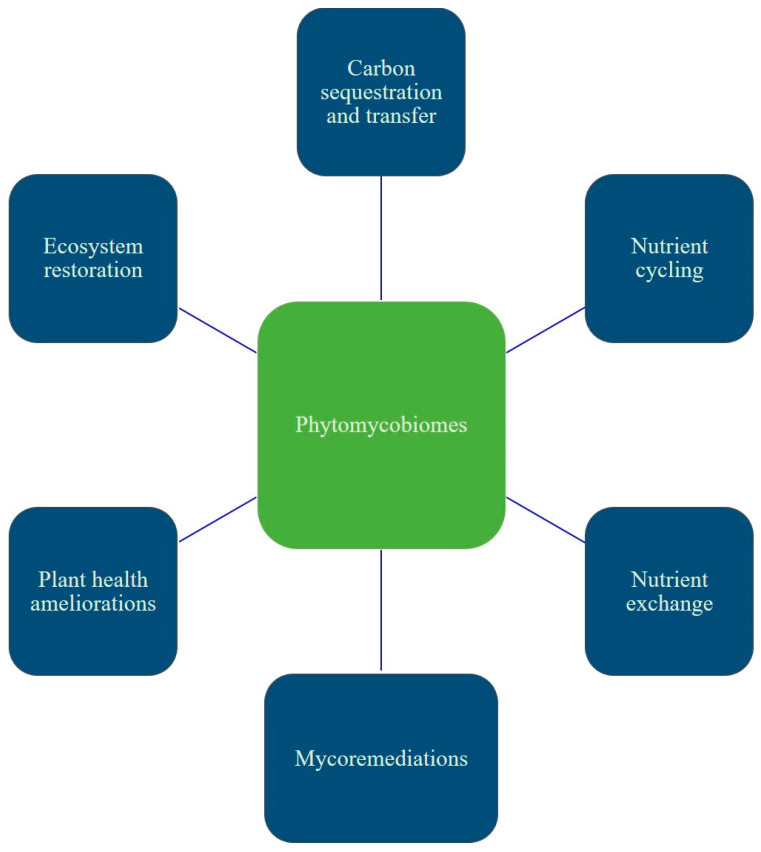
This figure summarizes how phytomycobiomes contribute to essential ecological functions such as nutrient acquisition, stress tolerance, soil health and carbon cycling.

Phytomycobiomes, chiefly, consist of the following principal functional groups: saprotrophs, pathogens, endophytes and several types of mycorrhizal fungi, including ectomycorrhizal (EcM), AM, ericoid and orchid associations [13,14]. Saprotrophs decompose dead organic matter into nutrient components [15], pathogens invade and damage living tissues, including all parts of the plant [16], endophytes grow within the plant and cause no symptoms [17] and mycorrhizal groups of fungi have symbiotic relationships with plant roots [18]. All of these make different contributions to the ecosystem’s functioning. Notably, saprotrophic fungi are the major decomposers in terrestrial ecosystems, facilitating the mineralization of organic residues with consequent turnover of nutrients [19]. Pathogenic fungi manipulate the fitness and population dynamics of their host to create major effects on the coevolution of plant communities [20]. Although asymptomatic, endophytes enhance the fitness of their hosts during environmental and biotic stress by altering the metabolic and signaling pathways of hosts [21]. Similarly, mycorrhizal symbioses are one of the most important forms of enhancing productivity and biodiversity in plants by mobilizing nutrients via molecular coordination through small RNAs and effector proteins [22]. Functionally, the overall function of the ecosystem is mediated by the activity of the multiple groups of fungi. To support this, we organize these fungal functions into four primary categories (mobilization of nutrients, stress mitigation, carbon sequestration, and suppression of pathogens) in keeping with the most significant physical processes through which phytomycobiomes support the dominant function of ecosystems. Important enzymes such as carbohydrase, phosphatase, protease, chitinase, and peroxidase help catalyze the carbon and nitrogen turnover by functioning in a different manner than they do in bacteria [23,24]. These enzyme-driven processes principally support the mobilization of nutrients, one of the vital highlighted functions. Plant resilience is enhanced, as these enzymes facilitate degradation for maximum mineral acquisition and distribution and maximum water absorption [25], which helps plants manage stress more effectively. Fungi also assist in maintaining ecological balance through parasitism, nutrient competition and the production of different bioactive organic compounds that overall sustain fungal diversity [26,27,28]. These antagonistic and competitive relations support pathogen suppression, which helps in the protection of plants by reducing disease pressure. Current understandings have improved due to molecular advances, which have enhanced our capacity to detect a wide range of fungi, providing valuable insight into host–microbe associations [29]. In addition, saprotrophic and mycorrhizal fungi contribute considerably to carbon sequestration, regulate decomposition and stabilize organic matter in soil ecosystems. However, the contributions of fungi to nutrient acquisition, soil health ameliorations and stress alleviations are less thoroughly documented than those of bacteria [30]. We believe that this gap in research arises from a long-standing bias towards only bacterial microbiome research, which has slowed down progress in appropriately understanding fungal functions. Although High-Throughput Sequencing (HTS) and multi-omics techniques have disclosed considerable fungal diversity, they continue to offer new insights about the relationship between community structure and different ecosystem functions [31]. By framing phytomycobiome activity within these four categories, this review links fungal diversity to measurable ecosystem services, and emphasizes the central theme of their ecological significance. This review accounts for a conceptual framework (Figure 1) that associates fungal diversity, important mechanisms and overall ecosystem outcomes, and deciphers molecular approaches that help characterize phytomycobiomes and summarize their pros and cons (Table 1), and also provides a crucial perspective on their actual usage in phytomycobiome exploration. This review also presents the comprehensive knowledge on phytomycobiomes and their functional roles in ecosystem sustenance (Table 2; Figure 2).

## 2. Phytomycobiome Diversity

Phytomycobiomes comprise a wide range of fungal communities and also represent one of the largest microbial groups associated with terrestrial ecosystems [32]. This group of organisms includes beneficial, neutral, and pathogenic species that reside in some regions of the rhizosphere, phyllosphere, and endosphere [33]. Modern DNA sequencing discloses that fungal diversity ranges from 2.2 to 3.8 million to over 11 million species, although only about 150,000 species are formally documented [34,35], and around 8000 species of fungi and oomycetes are known to damage plants’ well-being [36]. Broader investigation reveals the number of fungi to be between 0.5 and 10 million, with 1.5 to 5 million considered the most reliable estimate [37]. In addition, recent findings suggest the existence of up to 1.0 trillion microbial species worldwide; if even 1.0% were fungi, their total diversity would significantly exceed current estimations [38]. Thus, a true scale of global fungal diversity remains an open question for debate, which will require integrative research that combines taxonomy, ecology, and genomics to capture the full extent of the fungal biosphere.

Phytomycobiome diversity, particularly that of mycorrhizal fungi, as demonstrated by global EcM surveys, often increases toward higher latitudes [39]. However, depending on geographical context and classification thresholds, fungal diversity may also show a unimodal pattern, peaking at mid-latitudes rather than changing linearly [40]. Although climate and dispersal constraints shape fungal biogeography, its large-scale ecological patterns remain poorly understood. We recognize that plant-associated fungi show wide functional diversity and adopt several nutritional strategies. Mycorrhizal fungi form one of the major symbiotic groups and occur in most plant taxa. About 74% of plant species associate with AM fungi of the Glomeromycota, while EcM, orchid and ericoid types occur in about 2, 9 and 1% of plants, respectively [41,42]. Some plant species, such as poplars and eucalypts, maintain dual associations with both AM and EcM fungi [43,44]. These associations illustrate the ecological adaptability and evolutionary complexity of plant–fungal relationships. Saprotrophic fungi drive decomposition and nutrient turnover, sustaining plant succession and soil stability. Ascomycetes and Basidiomycetes, including over 8500 Basidiomycete species, degrade lignocellulose in forests and grasslands [45,46,47]. Molecular data reveal greater diversity than culture-based methods, and elevated atmospheric CO_2_ may reduce soil carbon by ~50% through enhanced fungal activity [48]. Despite considerable attention, the adaptive capacity of fungal networks under different environmental conditions remains inadequately resolved. It seems that an integrated framework approach combining molecular, ecological and evolutionary perspectives may clarify the functional role of fungal assemblage and enhance the predictive consideration of ecosystem resilience [49]. Thus, fungal diversity supports multiple mechanisms that, together, shape ecosystem performance. Distinct taxa supply complementary enzymes that release nutrients, stabilize soil carbon and restrict pathogen activity. These mechanisms do not act in isolation; they converge to create robust ecosystem services. Diversity, therefore, serves as the foundational unit for the functional capacity of fungi (Figure 1).

## 3. Molecular Techniques in Phytomycobiome Analysis

Molecular approaches have fundamentally reshaped the study of fungal communities, offering new perspectives on ecological patterns and biodiversity. The use of Sanger sequencing on PCR amplicons in the 1990s marked the inception of molecular fungal ecology, enabling the identification of fungi directly from environmental samples [50]. More recently, HTS has greatly expanded both taxonomic breadth and resolution, allowing researchers to detect a broader range of taxa and estimate their relative abundance with greater precision [51]. While these methods have considerably advanced our understanding, we would note that interpretation of sequence data still requires caution, given potential biases in amplification and database limitations. To structure the following technical approaches, we group techniques into five domains: (A) fingerprinting/community-profile methods that rapidly indicate community shifts (RAPD, T-RFLP, DGGE, SSCP, ARISA); (B) targeted molecular assays for specific taxon detection and quantification (DNA barcoding, qPCR); (C) HTS approaches that resolve community composition at species or OTU levels (DNA/RNA metabarcoding, NGS); (D) omics-based methods that reveal functional potential (metagenomics, metatranscriptomics, proteomics, metabolomics); and (E) deep learning and analytical approaches. This structure clarifies purpose, resolution and typical applications, which subsequently informs the selection of an appropriate method for phytomycobiome characterization. Furthermore, Table 1 summarizes the comparative performance of key phytomycobiome characterization techniques, and also highlights their optimal applications, resolution, quantitative capacity and major methodological biases to enable the selection of the most appropriate technique.

### 3.1. Fingerprinting/Community-Profile Methods

#### 3.1.1. Random Amplified Polymorphic DNA (RAPD)

RAPD investigation practices short, arbitrary primers to amplify anonymous regions of fungal genomes, which produce different banding patterns that reveal strain-level variation and host-related diversity [52,53]. This technique does not depend on prior sequence information; we find it to be specifically suited to pilot surveys of genetic diversity where reference data are inadequate [54]. RAPD has the ability to clarify both taxonomic and ecological patterns. Studies on *Thielaviopsis paradoxa* from South America, for example, reported largely clonal populations when RAPD profiles were compared with ITS data [55]. The method has also been applied for diagnostic resolution in *Malassezia* fungi [56], and for the identification of genotypic variation in *Metarhizium anisopliae* and *Beauveria bassiana* from soil, revealing its potential for assessing indigenous strains [53]. Although its reproducibility varies with reaction conditions, RAPD is considered a preliminary technique for mapping fungal diversity before applying more sequence-specific approaches. Thus, RAPD provides a rapid, low-cost fingerprint of genetic variability but suffers reproducibility issues, making it most suitable for preliminary diversity screening rather than fine taxonomic assignment.

#### 3.1.2. Terminal-Restriction Fragment Length Polymorphism (T-RFLP)

T-RFLP relies on the digestion of fluorescently labeled PCR amplicons with restriction enzymes, followed by electrophoretic separation, where only terminal fragments are visualized [57,58,59]. In our view, the technique remains a reliable approach for profiling fungal communities, because fragment size and signal intensity from digested ITS regions yield reproducible fingerprints reflecting taxon-specific sequence variation [60,61]. However, the discriminatory ability of this method largely depends on enzyme selection that influences both the resolution and the accuracy of fungal identification [59]. T-RFLP has been used successfully to study different fungal communities in plant and soil systems. For instance, it has helped to recognize yield decline in oilseed rape monocultures, where a dense pathogen-causing population was linked to constant cropping [59,62]. T-RFLP yields community profiles useful for detecting compositional shifts; however, it delivers low taxonomic resolution and can conflate distinct taxa with identical fragment sizes.

#### 3.1.3. Denaturing Gradient Gel Electrophoresis (DGGE)

DGGE separates PCR amplicons of equal length but distinct sequences through the differences in their melting behavior within a denaturant gradient, enabling the detection of single nucleotide polymorphisms and host-specific fungal strains [52,63]. The method, grouped with Temperature Gradient Gel Electrophoresis, detects sequence polymorphisms based on DNA duplex stability rather than enzyme restriction sites [64]. Originally developed for microbial community studies, DGGE now serves widely in environmental and rhizosphere analyses, where individual bands can be excised and sequenced for taxonomic identification [65]. Applications in plant-associated fungal ecology, including the strawberry and oilseed rape rhizospheres, have revealed species-specific community structures, though fungal profiles often display higher variability than bacterial ones [63,66]. Despite its value for visualizing dominant taxa and profiling uncultured fungi directly from environmental DNA, DGGE provides limited sensitivity and comparability between gels [64,67]. DGGE offers rapid visual profiling of dominant fungal community patterns, but its low sensitivity and poor taxonomic resolution limit its usefulness for comprehensive mycobiome analysis.

#### 3.1.4. Single-Strand Conformation Polymorphism (SSCP)

SSCP enables the separation of ssDNA fragments with the help of their secondary structures, facilitating the detection of sequence polymorphisms and point mutations with relatively simple laboratory requirements [59,63,68]. In contrast to DGGE or T-RFLP, the absence of GC clamps, gradient gels, and restriction digestion makes SSCP less technically demanding and more economically feasible [69]. Overall, we identified this technique as being important for profiling complex mycobiomes such as *Ophiocordyceps sinensis*, where it can disclose rare taxa representing less than 0.2% of the entire fungal community [69]. The method also provides rapid and cost-efficient insights into the rhizosphere community, including low-diversity *Trichoderma*-dominated soils that have a considerable influence on the functioning of the soil [59]. Despite these advantages, SSCP appears less informative than sequencing-based approaches and may serve best as a preliminary or comparative tool in large-scale ecological surveys [59,69]. Finally, SSCP can detect subtle sequence variations within fungal communities, but its low throughput and incomplete taxonomic determination confine its value for comprehensive mycobiome analyses.

#### 3.1.5. Automated Ribosomal Intergenic Spacer Analysis (ARISA)

ARISA provides a rapid alternative to culture-based community assays by adapting the earlier RISA framework [70]. Rather than sequencing, the method relies on fragment length polymorphisms within the ITS region, which often separate taxa to the genus or, at times, species level [71,72]. Fluorescently labeled primers such as ITS-1F/ITS-4 or 2234C/3216T are used to amplify ITS1 or ITS2, with subsequent capillary electrophoresis-generating profiles that act as population fingerprints [73,74,75]. Specialist primers remain available for narrower groups, including obligate anaerobes [76]. The main strength of ARISA lies in its handling of soils and decaying plant tissue, where isolation yields are low [77,78,79]. Forest pathologists have advanced this approach further by detecting phytopathogens directly from leaves or bark without culturing, which remains a practical advantage over sequencing-based platforms [80,81]. The method is not without ambiguity, given that fragment size similarity can mask taxonomic distinction, yet recent protocols increase resolution by combining ITS with IGS, RNA polymerase II, or β-tubulin markers [82,83,84,85]. We still question its ability to standardize abundance estimates across laboratories, although its speed and low cost ensure its continued use where HTS is impractical. ARISA provides rapid, high-throughput community fingerprints, but its lack of taxonomic resolution and sensitivity to PCR biases limit its usefulness for detailed phytomycobiome characterization.

### 3.2. Targeted Molecular Detection and Quantification

#### 3.2.1. DNA-Based Molecular Markers and Barcoding

Conventional molecular identification and DNA barcoding differ primarily in that barcoding targets a standardized genetic region specific to a taxonomic group, while conventional systems may use many genetic regions depending on the organism and objective [86]. In fungi, the ITS of nuclear ribosomal DNA, comprising ITS1, 5.8S, and ITS2, has emerged as the universal barcode, which is widely used in environmental DNA studies [86,87]. ITS1 and ITS2 are favored due to their high interspecific variability, conserved primer sites, and multiple genomic copies, making them suitable for metabarcoding and amplification from low-quality DNA [88]. Many studies quantify diversity through OTUs, which act as sequence-based proxies for taxa and underpin measures of richness, frequency, abundance, and distribution [87,89]. Molecular surveys typically employ either targeted metagenomics, which amplifies specific markers via PCR, or shotgun metagenomics, which sequences all genetic material in a sample [88]. These approaches are crucial for assessing fungal community composition, structure, and ecological function, though we would caution that PCR biases and database limitations can influence interpretation. Barcoding gives species-level identification when reference libraries exist; its accuracy is considerably influenced by marker selection and reference completeness.

#### 3.2.2. Real-Time Quantitative PCR (q-PCR)

q-PCR remains one of the most pragmatic tools for estimating fungal biomass from environmental DNA without culture bias, although its reliability rests heavily on primer choice [90,91]. Broad-range assays often target conserved 18S regions, while finer discrimination relies on ITS or single-copy markers such as β-actin, yet no single locus offers a universal balance between coverage and specificity [90,92]. Primer sets such as FR1/FF390 are frequently cited as a practical compromise, although performance can vary among different substrates. Reported Ct values for *Fusarium*-infected barley tissue range between 26 and 30, compared with 36 in healthy samples, which suggests good sensitivity but says little about absolute quantification across different matrices [93]. In our view, q-PCR is appropriate as a comparative rather than an absolute quantification tool. It works most reliably when applied to examine temporal or treatment-based contrasts, provided that appropriate standards and inhibition controls are included in the probe [92,94]. qPCR provides high sensitivity for known targets, but it necessitates species-specific analyses and can not profile unknown diversity.

### 3.3. High-Throughput Sequencing

#### 3.3.1. DNA-Based Metabarcoding

DNA metabarcoding provides a practical means for the detection of fungal taxa without culturing through targeting barcode regions within environmental DNA and sequencing them at a desired scale [95,96]. In this case, our preference is to use the ITS, yet we remain cautious while interpreting ITS1 and ITS2 outputs because primer selection can affect the perceived diversity [97,98]. ITS1 frequently resolves Basidiomycota more accurately, although it appears vulnerable to bias introduced during the amplification process, while ITS2 typically captures a broader range of taxa due to its more conserved priming regions [99,100]. Metabarcoding now faces fewer challenges from sequencing capacity; however, the progress of this technique is primarily limited by database quality. UNITE is the default reference for ITS classification, yet it still contains many gaps and occasional misannotations, a concern that needs to be adequately addressed [30,72,101]. Thresholds for genus- or family-level matching may offer some consistency, though confidence in species ranking is sometimes misplaced when dealing with sparse or unevenly sampled lineages [101,102]. Despite these constraints, metabarcoding has produced lineages unlikely to appear in culture-based work, including some that are early-diverging groups of ecological significance [96]. Long-read platforms may eventually clarify relationships within complex genomes, although high costs and error rates currently restrict their use to exploratory rather than routine analysis [101,103]. DNA-based metabarcoding enables broad, high-resolution profiling of fungal communities, but primer bias and variable marker performance can distort their relative abundance and taxonomic representation.

#### 3.3.2. RNA-Based Metabarcoding

RNA-based metabarcoding has been promoted as a means of distinguishing metabolically active fungi from background DNA noise, on the premise that transcribed genes provide a closer proxy for living biomass [71,80,85,104]. In contrast to DNA surveys, which record relic material long after cell death, RNA-based protocols restrict analysis to environmental RNA and therefore offer a narrower, arguably more functional, view of the mycobiome [105]. The workflow still relies on extraction, reverse transcription to complementary DNA, and sequencing, yet its outputs tend to align more closely with organisms considered active at the time of sampling [85,106]. We would argue that this selectivity is both a strength and a liability, since exclusion of dormant propagules risks underestimating latent inoculum capable of rapid reactivation [106]. Reported studies have nonetheless shown clearer detection of symbiotic Glomeromycota in grassland soils and increased representation of wood-decaying taxa in decomposed substrates, patterns less apparent in DNA-based datasets [105]. Some ascomycetes, such as *Saccharomyces* and *Komagataella*, also appear more resolvable when ITS1 is applied to RNA-derived material [85]. A cautious integration of RNA and DNA evidence seems preferable in practice; the former reflects activity, the latter establishes presence, and neither alone offers a complete ecological account [107]. RNA-based metabarcoding captures the active fraction of the fungal community with enhanced functional relevance, but RNA instability and rapid turnover can introduce bias and obscure quantitative understanding.

#### 3.3.3. Next-Generation Sequencing

HTS, or next-generation sequencing (NGS), has substantially expanded its capacity to study fungal communities on a large scale [108]. Early platforms, such as 454 sequencing, enabled the characterization of fungal assemblages from environmental DNA, while modern NGS methods have deepened our understanding of soil- and root-associated fungi [108,109]. Surveys often detect extensive OTU richness; for example, hundreds of fungal OTUs per plant root system and hundreds of thousands of prokaryotic OTUs in global analyses [109,110]. Amplicon-based HTS generally targets barcode regions such as ITS to identify taxa in mixed samples, whereas shotgun sequencing examines whole-community DNA, but may struggle with low-abundance fungal genomes and complex structures [100,111,112]. The current ground-breaking research in metabarcoding metagenomics and HTS has accurately unraveled fungal taxonomy, diversity and their ecological roles in ecosystem functioning [107,113]. In particular, HTS and omics are of central importance for studying a wide range of fungi, including uncultivable or cryptic fungi in forest ecosystems, revealing community organizations and ecological functions that traditional methods often overlook [114,115]. HTS provides comprehensive, high-resolution profiles of phytomycobiome communities. We would note, nevertheless, that technical biases and uneven sequencing depth remain important limitations when interpreting these data.

### 3.4. Omics and Functional Approaches

#### 3.4.1. Metatranscriptomics

Metatranscriptomics, a very unique technique, provides a functional view of phytomycobiomes by allowing environmental RNA to be sequenced instead of residual DNA, allowing active populations to be separated from background genetic material [99,116]. In principle, it minimizes the amplification biases inherent to PCR and widens taxonomic resolution through access to non-ribosomal genes, although such entitlements remain difficult to benchmark [117,118]. The strongest utility has been reported in systems where conventional amplicon surveys reach their interpretative limit, particularly when distinguishing symbionts from saprotrophs or latent pathogens [105,119,120]. In Norway spruce, shifts in carbohydrate and amino acid metabolism are attributed to root-associated *Cortinarius*. Grapevine dieback assays reveal *Eutypa lata* as the dominant agent within complex infections previously hidden by isolation-based diagnostics [119,120,121]. Forest litter work indicates phylum-level richness with contrasting degradation profiles among EcM and saprotrophic fungi, and agricultural trials link monoculture stress and cover crop adoption to marked changes in the expression of lignocellulose-degrading enzymes [114,122,123]. Continuous *Morchella* cultivation appears particularly detrimental to soil fungal diversity, though fallow periods partly restore activity [124,125]. Interpretation of metatranscriptomics remains hindered by sparse reference genomes and limited annotation depth, leaving a sizeable fraction of transcripts functionally unresolved [117,126]. Even so, pairing presence with activity remains, in our view, its most persuasive contribution to fungal ecology. Metatranscriptomics reveals the actively expressed functional genes of fungal communities with significant ecological relevance, but RNA instability decreases data reliability and increases technical complexity.

#### 3.4.2. Metabolomics

Metabolomics quantifies fungal metabolites under 1500 Da and remains the most direct route to inferring functional state rather than genetic potential [107,127]. Mass spectrometry is still the analytical backbone, especially when paired with chromatographic separation to discriminate closely related taxa [128,129]. The cogent document belongs to diagnostic case studies: *Aspergillus* sp., for instance, show different markers like the tetrapeptide Leu-Glu-Leu-Glu, while Candida albicans secretes characteristic volatomes enriched in 3-methyl-2-butanone and styrene [130,131,132]. An extensive and comparative survey exhibits relatively stable intra-species metabolite profiles but clear divergence between species, which implies an evolutionary signal instead of random variation [133]. Natural product research has capitalized on this, uncovering novel compounds such as 7-desmethylcitreoviridin from *Aspergillus terreus* [134,135]. NMR- and LC-MS-based chemometrics further support the use of metabolite signatures for taxonomic inference, although we remain careful about over-interpreting profiles that may shift with culture conditions [136,137]. The approach is undoubtedly powerful, but still requires firmer standardization before classification claims can be made with confidence. Metabolomics provides direct insight into the biochemical outputs and functional states of fungal communities, yet metabolite complexity, environmental variability, and difficulties in linking metabolites to specific taxa limit precise interpretation.

#### 3.4.3. Proteomics

Proteomics, now integral to fungal omics, enables the examination of proteins expressed under defined conditions and has proved useful across pathogenicity research and systematics through fingerprinting and biomarker discovery [107,138,139,140]. MALDI-ToF-MS is one of the more practical advances, offering rapid species identification from intact cells in genera such as *Aspergillus*, *Fusarium* and *Trichoderma* at a lower cost than sequencing, though the reliability of classification still hinges on database completeness [140]. LC-MS/MS provides deeper resolution, including detection of extracellular vesicle proteins implicated in infection processes, while MALDI-based resistance profiling offers a swift method for screening antifungal tolerance, though largely confined to drugs with well-characterized proteomic signatures [141,142,143]. Metaproteomics extends this approach to soil communities by linking enzyme activity with taxonomic origin through pipelines, such as “PROteomics result Pruning & Homology group ANotation Engine” (PROPHANE), yet protein diversity in environmental samples often hampers clear functional attribution [144]. Unlike metagenomics, which identifies genetic potential, metaproteomics captures real-time activity and has been used to explain niche exclusion phenomena such as truffle brûlé [145]. It is particularly valuable for obligate biotrophs that cannot be cultured; in *Blumeria graminis*, for example, stage-specific protein sets are resolved during barley colonization [146]. Secretome analysis continues to expose virulence determinants, including the first identified root-invading effector, although functional relevance in planta is not always straightforward to confirm [138]. Proteomics offers direct evidence of the functional proteins produced by fungal communities, but low biomass, complex sample preparation, and complexity in naming proteins to specific taxa challenge its resolution and interpretability.

#### 3.4.4. Metagenomics

Metagenomics enables recovery of total DNA directly from environmental samples without culturing, which provides a direct view of fungal diversity within mixed communities [108,113,147]. Two main strategies are commonly used, although they differ in resolution and cost. Targeted sequencing of barcode regions, most often the ITS locus, remains widely chosen because it is affordable and sensitive, although it is affected by primer bias and uneven amplification across taxa [71,111,148]. The ITS region now functions as the accepted barcode for species-level identification and is routinely compared with reference databases such as UNITE, although database completeness determines how confidently sequences can be assigned [51,97,98,112,114,149]. Broader environmental surveys report substantial richness across several phyla, although observed correlations with factors such as elevation and soil chemistry may not necessarily indicate functional causation [114,150]. Metagenomics demonstrates functional gene content and may identify unculturable taxa, but it needs higher sequencing depth, complex assembly, and has issues linking genes to specific taxa.

### 3.5. Deep Learning and Analytical Approaches

Deep learning (DL) and analytical approaches have nowadays become instrumental in the understanding of phytomycobiomes within ecosystem services [151]. DL, particularly convolutional neural networks (CNNs), automatically extracts the features from complex biological data and has emerged as one of the most powerful tools for unraveling the plant–microbiome complex [152,153]. Distinguished by neural networks with three or more layers, DL receives its name from the depth of its architecture, with multiple hidden layers providing these algorithms with the capability to occasionally surpass human performance in some cases due to the extraordinary precision accomplished by these computational stages [152,154]. DL has primarily transformed taxonomic classification and functional analysis in plant–microbiome research through its superior pattern recognition capabilities and automatic feature learning [155]. CNN models have revealed a unique performance in specific applications, with multi-layer CNN architectures achieving 97.13% accuracy in the classification of fungal-driven diseases in mango leaves [156]. Semantic segmentation approaches using UNet-CNN architectures have proven specifically effective for quantitative assessment of plant disease, achieving 96.08% mean pixel accuracy in the identification of powdery mildew-affected areas in cucumber [157]. DL models consistently outperform classical machine learning (ML) processes in plant–microbiome research, with CNNs achieving 92–99% accuracy [158,159] compared to 69–84.91% [160,161] for traditional methods. However, accuracy performance gaps exist between controlled laboratory conditions (95–99%) and real-world field deployment (70–85%) [162]. Thus, traditional ML approaches remain constrained by their dependence on domain-specific feature engineering and limited ability to generalize across a wide range of environmental conditions, while DL models require dataset sizes to be expanded and imaging conditions to be diversified to further enhance their robustness.

**Table 1:** Comparison of molecular and analytical techniques used in phytomycobiomes characterization.

Techniques	Target	Resolution (Taxonomic/Functional)	Quantitative	Best Application	Main Limitations	Efficacy	Reference
ITS Amplicon Sequencing	rDNA	High taxonomic resolution (species or genus level)	Semi-quantitative (gives relative abundance)	Profiling fungal communities in soil, roots, or plant tissues; studying diversity and composition.	PCR bias, uneven amplification, limited to known reference databases, not functional information	Highly effective method for fungal community profiling	[163]
DNA-based metabarcoding	Specific barcode genes such as 16S rRNA (bacteria), ITS (fungi), COI (animals), rbcL/matK (plants)	High taxonomic resolution (species or genus level); Functional—indirect (depends on taxonomic inference)	Semi-quantitative (provides relative abundance)	Profiling biodiversity in environmental samples (soil, water, plant tissues); studying community composition and shifts	PCR and primer bias; incomplete reference databases; cannot infer true function or abundance.	High-throughput, sensitive community profiling.	[164]
ARISA	Ribosomal Intergenic Spacer Region (ITS between 16S and 23S rRNA genes in bacteria or 18S–28S in fungi)	Moderate (mainly community-level or species richness)	Semi-quantitative (estimates relative abundance based on fragment peak intensity)	Comparing microbial community structure, diversity	PCR and fragment length bias; limited resolution for complex communities	Moderately effective for taxonomic resolution.	[165]
DGGE	PCR-amplified fragments of 16S rRNA (bacteria) or 18S/ITS regions (fungi)	Moderate (genotype or species-level)	Semi-quantitative (shows relative abundance by band intensity)	Comparing microbial community composition, detecting dominant species, and monitoring community shifts across treatments	Limited to dominant taxa; co-migration of different sequences; low sensitivity for rare species; labor-intensive.	Gel-based moderately effective for taxonomic resolution	[166]
Metabolomics (GC-MS, LC-MS)	Small fungal and plant metabolites (organic acids, amino acids, secondary metabolites, VOCs) produced during plant–fungus interactions	Functional—reveals metabolic pathways, stress responses, and signaling molecules	Quantitative and qualitative (detects and measures metabolite concentrations)	Understanding fungal metabolic profiles, plant–fungus interactions, symbiosis or pathogenesis, and functional diversity within phytomycobiomes	Expensive instrumentation; data analysis requires expertise; may miss low-abundance or volatile metabolites	Functional metabolite profiling without direct taxonomic resolution.	[167]
Metagenomics (Shotgun sequencing)	Total community DNA from plant tissues, rhizosphere, including fungi	High taxonomic (species or strain level) and high functional (genes, enzymes, metabolic pathways)	Semi-quantitative to quantitative (gives relative gene abundance; can be normalized for more accurate quantification)	Comprehensive profiling of fungal community composition, functional genes, secondary metabolite biosynthetic pathways, and plant–fungus interaction networks	Expensive; complex data analysis; requires high-quality DNA; databases may lack full fungal genome coverage	Comprehensive, untargeted genome-wide profiling with functional insights.	[168]
Metatranscriptomics	Total community RNA (mRNA) from plant tissues, rhizosphere	High functional resolution (gene expression, metabolic pathways) and moderate taxonomic (depends on annotated transcripts)	Measures gene expression levels and relative transcript abundance	Studying active fungal communities, plant-fungus interaction dynamics	RNA is unstable; requires immediate preservation; high computational demand; reference databases for fungi are limited	Effective in functional and active gene expression	[169]
Proteomics (LC-MS/MS)	Proteins and peptides produced by fungi, plants, and other microbes in the community	High functional resolution—identifies metabolic and signaling proteins; moderate taxonomic—depends on protein database coverage	Quantitative and qualitative (measures protein abundance and identifies differentially expressed proteins)	Understanding functional activity, enzyme secretion, defense responses, and symbiotic or pathogenic interactions within phytomycobiomes	Complex sample preparation; limited fungal protein databases; difficulty in detecting low-abundance proteins; expensive instrumentation	Functional protein data, technically high in demand.	[170]
qPCR	Specific DNA sequences or genes	High taxonomic (species or genus level when primers are specific); Functional (when targeting particular genes)	Quantitative (measures absolute or relative gene copy numbers)	Detecting and quantifying specific fungal taxa or functional genes; monitoring biocontrol agents or pathogen load in plant–soil systems	High sensitivity to inhibitors; only detects targeted organisms/genes, not the whole community	Effectively quantifies the targeted gene	[171,172]
RAPD	Random segments of genomic DNA	Taxonomic: Moderate—differentiates at species or strain level	Qualitative; shows genetic variation patterns but not abundance	Assessing genetic diversity, strain differentiation, and population structure of fungal isolates from plant–soil environments	Low reproducibility; sensitive to reaction conditions; dominant marker (cannot distinguish homozygotes and heterozygotes); limited for mixed communities	Highly effective in revealing genetic polymorphism.	[173,174]
RNA-based metabarcoding	Ribosomal RNA (rRNA) or cDNA from reverse-transcribed RNA (e.g., ITS, 18S rRNA, 16S rRNA) representing metabolically active fungi and microbes	High taxonomic resolution (species/genus level for active taxa); Functional—indirect (reflects living, active community)	Semi-quantitative (relative abundance of active microbial members)	Identifying active members of phytomycobiomes; distinguishing live vs. dormant fungal populations; studying dynamic responses to stress or infection	RNA degrades easily; results depend on RNA stability and extraction efficiency	Effective in living, active biodiversity	[171,175]
SSCP	DNA sequence variation (usually in ribosomal or marker genes)	Taxonomic (species or strain level)	Qualitative method	Used to detect genetic variation among microbial, including fungal communities	Limited to detecting single-base changes in short fragments. Less effective for complex or diverse samples.	Effective in analyzing genetic variation through single-point mutation	[176,177]
T-RFLP	DNA—usually 16S rRNA or ITS regions amplified by fluorescently labeled PCR primers	Taxonomic (genus to species level)	Semi-quantitative (relative abundance inferred from fragment peak intensity)	Comparing microbial or fungal community composition across samples; monitoring community shifts in phytomycobiomes and soil microbiota	Cannot identify taxa directly (requires database matching); different taxa can share fragment sizes; limited detection of rare taxa	Effective use in comparative profiling of community structure	[178,179]

## 4. Phytomycobiomes and Their Contribution

### 4.1. Nutrient Cycling

AM fungi, by forming extensive hyphal networks, act as primary agents of nutrient dynamics within terrestrial ecosystems that reach beyond the rhizosphere, and improve plants’ ability to access water and nutrients [180,181,182]. The special hyphae invade fine soil pores that are inaccessible to roots and enable the uptake of otherwise immobile phosphorus and other important micronutrients [183]. Nutrient exchange takes place through specialized arbuscules, which create interfaces between the host and fungi [184]. These interactions represent a significant component of ecosystem carbon flow, with about 13% of host-assimilated carbon allocated to EcM fungi and 6% to AM fungi [185]. In return, AM fungi meet nearly all of a host’s phosphorus demand and one-fifth of its nitrogen requirement, whereas EcM fungi may supply up to 70% of phosphorus and 80% of nitrogen needs [186,187]. However, environmental factors, fungal and host identity, and microbial competition influence the nutrient exchange efficiency [188,189,190,191]. AM fungi also assist with soil nutrient mobilization with the secretion of solubilizing enzymes, such as phosphatases, that release phosphorus from organic and inorganic compounds [192,193]. They enhance zinc acquisition ability in cereals, providing up to 24% of shoot zinc in wheat and 13% in barley [194]. The hyphal networks also interconnect to neighboring host plants that support horizontal nutrient transfer within plant communities [195]. Additionally, AM fungi influence soil structure, overall health and microbial balance as they secrete glomalin, which helps strengthen soil aggregates, organic acids that mobilize metals and also helps in the interactions that promote bacteria-led nutrient mineralization [183,193,196].

### 4.2. Carbon Cycling

Fungi play a central role in terrestrial carbon cycling by mediating both carbon release and carbon storage (Figure 2). Saprotrophic fungi, on the other hand, play a crucial role in decomposition, which converts complex organic molecules into accessible nutrients, including carbon for plants and other organisms [197,198]. This stabilizing capacity is especially pronounced in EcM fungi, whose mycelia, biofilms, and fruiting bodies are enriched in recalcitrant compounds that decompose slowly, producing a persistent “entombing effect” [199] that routes large quantities of microbial residues into long-lived soil carbon pools. EcM fungi also influence carbon cycling through nutrient acquisition mechanisms. They directly mine organic nitrogen using important extracellular enzymes, which reduces their reliance on saprotrophic decomposition and slows organic matter turnover [200,201]. In contrast, AM fungi depend on saprophyte-mediated mineralization before accessing nutrients, a process that accelerates carbon loss [202]. This functional divergence is reflected in ecosystem responses: EcM-associated plants overcome nutrient constraints more effectively, exhibit greater climate tolerance, and show substantially higher growth responses, approximately 30% biomass increase under elevated environmental CO_2_ conditions. AM-associated species, by comparison, display little to no biomass change and contribute less to long-term carbon stabilization due to their faster hyphal turnover [203]. The entombing effect of EcM fungal tissues, their enzymatic access to organic nitrogen, slower decomposition pathways, and the high biomass production of EcM-associated plants allow them to act as major terrestrial carbon reservoirs. This contrasts with AM-dominated systems and highlights the central role of fungal groups in shaping soil carbon persistence and broader terrestrial carbon cycling [204].

### 4.3. Crop Protection

Some fungi exist in nature that establish alliances with host plants that increase plant growth and protection without any pathogenic effects [205]. The contribution of plant hormones such as auxins, cytokinins and gibberellins appears instrumental to these interactions, influencing the uptake of nutrients, mineral solubilization and adaptive responses to biotic stressors [205,206]. In addition, there are some specific biomolecules that fungi release in response to biotic stressors to suppress further activities of plant pathogens. Among them, *Trichoderma* spp. have gained momentum as efficient biocontrol agents that release indole-3-acetic acid (IAA) and salicylic acids to stimulate root development and activate the defense mechanisms of their host plants [206,207]. In addition, a protective capacity arises through both direct antagonism and systemic induction of plant immunity. These functional groups of fungi produce antimicrobial metabolites that inhibit plant pathogens by more than 80%, usually through transcription factor activation, such as *WRKY* and *MYB*, which regulate acquired and induced systemic resistance [208,209,210]. However, such responses vary with fungal strain and prevailing environmental conditions. For example, gliotoxin remains comparatively stable under dry soil conditions, with soil pH having little influence on its persistence. Under wet and unsterile soil conditions, however, pH plays a major role: higher pH markedly accelerates gliotoxin degradation, whereas lower pH supports greater stability [211]. *T. asperellum* Q1 enhances the plant biomass of *A. thaliana* under an iron-deficient environment by producing siderophores that mobilize insoluble Fe and stimulate IAA accumulation in roots. Both the fungus and its siderophores increase endogenous IAA levels, which indicates a dual role in iron acquisition and hormone-mediated growth promotion [211]. This variability raises questions about reproducibility and the reliability of laboratory findings under field conditions. *Trichoderma* spp. enhances stress tolerance ability of plants through modulation of host-redox metabolism, maintaining equilibrium within the ascorbate–glutathione cycle and oxidative pentose phosphate pathway [212,213]. Biochemical defense molecules such as superoxide dismutase and catalase help maintain and stabilize cellular homeostasis, on the other hand, volatile compounds like terpenes and benzenoids contribute to defense signaling and herbivore deterrence [209,214].

Antagonistic activity in *Trichoderma* spp. arises from three important processes (mycoparasitism, antibiosis, and competition). Mycoparasitism involves the coiling of hyphae, appressorium formation, and hydrolytic enzyme production, including chitinases, β-1,3-glucanases, and proteases, which evade pathogen cell walls [215,216,217]. Antibiosis occurs through bioactive compounds like gliotoxin, trichokonins, peptaibiotics and harzianic acid [218,219]. The competitive atmosphere arising from quick growth and efficient nutrient confiscation further restricts pathogen proliferation [220,221]. Notably, *T. harzianum*, *T. asperellum* and *T. virens* achieve pathogen suppression between 44% and 92% against *Fusarium*, *Rhizoctonia* and *Sclerotinia* [222,223]. Moreover, *Trichoderma* spp. mediated biocontrol formulations demonstrate significant promise; their performance, however, remains context-dependent. These formulations should be validated under different agroclimatic conditions before they can substitute for chemical fungicides [224,225,226]. Suitable strains of fungal inoculants more broadly reveal a sustainable approach to improve soil fertility and crop resilience [227]. In addition, EcM fungi act as primary defenders in forests, suppressing pathogens through structural protection, enhanced host immunity, and competitive dominance, especially in woody plant root systems. Evidence from forest ecosystems consistently shows that EcM fungi function as primary antifungal agents [204]. EcM-dominated root systems experience lower pathogen incidence because the ‘Hartig’ net and surrounding hyphal mantle act as a protective armor, while EcM symbiosis also enhances host immune responses and reduces tissue susceptibility to necrotrophic fungi [228,229]. These combined structural, biochemical, and competitive advantages clarify why EcM fungi play a disproportionately large role in disease suppression, especially in woody perennial systems and forest soils. We expect that integrating them within crop rotations and cover-cropping systems supports fungal networks that enhance nutrient transfer, carbon storage, soil, and plant health [230,231,232].

### 4.4. Ecosystem Resilience

Fungal communities form an essential component of ecosystem resilience and serve as reliable indicators of soil recovery after disturbance (Table 2). The hyphal density of fungi exhibits how well the soil particles are aggregated together and how stable this structure is [233]. Thus, the functional role of fungi reveals a clearer picture in ecosystem restoration, which is mostly important for monitoring systems in which vegetation recovery proceeds gradually (Figure 2). This specific characteristic of fungi improves soil and plant health [234]. Moreover, mycorrhizal fungal hyphae link around 340,000 plant species with nearly 50,000 fungal taxa through mycelial bridges, which facilitate the exchange of water, nutrients, carbohydrates, amino acids and plant growth hormones [13,235,236]. These networks influence soil aggregation, biological, chemical and physical properties and hydrological balance, thereby improving community-level resilience to drought and temperature stress. The claims of plant “communication” through mycorrhizal hyphae remain speculative, but evidence supports their role in maintaining forest ecosystems and regeneration potential after climatic or anthropogenic perturbations [237,238]. The extraradical hyphae redistribute nutrients and water beyond the rhizosphere, and enhance resource sharing among plants and aid recovery in degraded or fragmented ecosystems [239]. Moreover, mycoremediation extends this ecological capacity by using the ability of fungal metabolism to degrade or immobilize pollutants, thereby reversing the compromised quality of water and soil. Fungal resilience under less nutrient, acidic or arid conditions makes them appropriate for restoration in degraded environments [240,241]. The contaminant removal takes place through biosorption, bioaccumulation, bio-precipitation and enzyme-mediated mineralization [242,243]. Mycelial invasion through soil pores enhances contact with different pollutants, releasing organic acids and chelator molecules that mobilize or precipitate heavy metals [244,245,246]. Some frequently occurring genera like *Aspergillus*, *Penicillium*, *Fusarium*, *Verticillium* and *Phanerochaete* show cogent binding and uptake abilities, with *Fusarium* and *Cladosporium* strains accomplishing mercury removal rates exceeding 90% [243,247]. White-rot fungi such as *Trametes*, *Pleurotus* and *Ganoderma* deter important pollutants like polycyclic hydrocarbons, dyes and phenols through ligninolytic enzymes such as laccase and manganese peroxidase [248,249,250,251]. Some important fungi like the *Pleurotus* and *Aspergillus* species have reduced pesticide and dye loads by 98%, while *Ganoderma lucidum* and *Agaricus bisporus* have significantly removed COD, NH_4_^+^ and heavy metals from industrial effluents [252,253,254]. These suggest that integration of mycorrhizal and saprotrophic fungi together into restoration frameworks may improve soil resilience, accelerate the degradation process and support the design of adaptive management systems for pollutant-affected environments.

### 4.5. Communication and Symbiosis

The communication between plant-fungi is a highly sophisticated network. The mycorrhizal fungi communicate with plants via wide array of compounds that enable underground signaling and recognition [255]. The strength of the signaling and nutrient transfer varies by plant, being most pronounced between related plants and weaker between unrelated plants or those of different mycorrhizal types [256]. The initial communication between plants–fungi takes place through important signaling molecules. The plant begins the molecular dialogs through a secretion of strigolactones in phosphorus-deficient environment [257]. This serves as a critical signal that indicates that the presence of the cohort plant is receptive to fungal colonization [258]. AM fungi differ in detecting strigolactones, undergo certain dramatic changes like spore germination, enhanced hyphal growth, and branching and activation of oxidative metabolisms [259]. The activation of this metabolism leads to the generation of energy, which is required for the organelle division, ATP production and modulation in gene expression that enable AM fungi to establish symbiosis [260]. Moreover, on their surface, the plant roots have a specialized protein chitin-binding lysin motif (LysM) as a receptor molecule. These receptor molecules identify the cell wall materials (lipochitoloigosaccharides) of fungi. When these materials diffuse to the receptor molecules of plants, they reveal their beneficial, rather than harmful, nature to the plant [261]. The signaling chemicals enter the host plants and carry out a chain of reactions. Such reactions include the rise and fall of calcium levels inside the cells, called calcium spiking. Such calcium signals help the plant activate a pathway called the common symbiosis signaling pathways. Subsequently, plants undergo certain genetic modifications, allowing the fungus to enter and form a peaceful and beneficial symbiosis [257,261]. Transport of significant molecules within these networks occurs through cytoplasmic streaming, diffusion, surface movement, and conduction along rhizomorphs [236,262,263,264].

**Table 2:** Functional groups of fungi and their contributions to ecosystem services, and the major environmental factors that considerably influence the shape of phytomycobiome diversity, with major techniques used in specific cases.

S. No.	Fungal Group	Findings	Drivers	Molecular Methods	Reference
1.	Saprophytic, symbiotic, pathotrophic	Maintained nutrient cycling and ecosystem balance	Soil pH, N, moisture, altitude	Illumina MiSeq sequencing	[265]
2.	AM, EcM, pathogen	Spatial and soil variables shaped diversity; trees affected structure	Spatial factors, soil properties	Pyrotag ITS2 sequencing	[266]
3.	Symbiotrophic, saprotrophic, pathotrophic	Distinct β diversity and networks across forests	Soil pH, SOC, TN, TP, N/P	Illumina MiSeq Sequencing	[198]
4.	Generalists, specialists	Mixed plantations supported generalists and stability	N, P, S	ITS metabarcoding	[267]
5.	EcM	Diversity peaked in tropics; strong endemicity	Climate, pH, Ca, P	Pyrosequencing, metabarcoding	[149]
6.	Saprophytic, mycorrhizal, macrofungi	Diversity varied by vegetation and topography	Vegetation, humidity, precipitation, wind velocity	DNA sequencing	[268]
7.	Pathogens, saprotrophs, endophytes	Diversity substrate-dependent; soil-hosted unique OTUs	Temperature, precipitation, pH	PCR	[269]
8.	EcM	Community shifts linked to forest management	C/N, pH	ITS rDNA pyrotag sequencing	[270]
9.	EcM, saprotrophic	Supported uptake, mineralization	C/N, litter, pH	DNA sequencing	[271]
10.	EcM, AM, saprophytic, pathogenic	Dominance shifts altered nutrient cycling	Wildfire, clear-cutting, logging	MiSeq ITS1, SSU rDNA	[272]

## 5. Conclusions and Future Prospects

Phytomycobiomes represent an essential yet insufficiently integrated component of ecosystems. Although taxonomic catalogs have expanded considerably, our functional understanding of these biomes remains scarce. Fungi contribute considerably to nutrient mineralization, plant health ameliorations, ecosystem resilience and stabilization. The functional role of fungi largely depends on soil properties, climate and land-use history, and their disturbance can alter cooperation or competition within the community in unpredictable ways. Moreover, fungal diversity remains phenomenal at shaping the host’s traits. Accurate identification of fungi and their functional roles in a wide array of ecosystems needs to be thoroughly investigated that will reveal a distinct pattern of ecosystem stability and functionality. HTS have revealed hidden fungal networks; however, most studies remain largely descriptive. Future progress depends on explicitly coupling taxonomy with function through trait-based and systems-level frameworks that connect community composition to measurable ecological outcomes. Integration of multi-omics datasets, isotopic tracing and manipulative field trials will help clarify causal mechanisms and strengthen predictive accuracy. Phytomycobiome usage offers opportunities to reduce agrochemical use, enhance nutrient capture and increase crop resilience under stress.

Future research should swing from descriptive profiling to mechanistic, integrative frameworks that clarify how phytomycobiomes shape plant health and ecosystem functioning. Priority should be given to multi-omics approaches that merge metagenomic, transcriptomic and metabolomic data to resolve active fungal contributors and their functional traits. Equally important is the development of interpretable DL models capable of revealing beneficial fungi and functional genes that underpin different ecological functioning. Integration of multi-source remote sensing with root-zone sensors to hyperspectral imaging may reveal real-time ecological mapping of how varied microclimate conditions, nutrient fluxes, and soil’s physical, chemical, and biological processes regulate phytomycobiomes dynamics. At the systems level, ecological networks that incorporate co-occurrence patterns of plant–mycobiomes and metabolite signaling should be coupled with decision-support systems. To address these issues, uncompromised funding, multidisciplinary research teams and robust strategy supports are needed to ensure that phytomycobiomes contribute directly to different ecosystem functioning.

## Data Availability

All externally sourced data are accompanied by appropriate citations.
